# Determinants for COVID-19 vaccine hesitancy in the general population: a systematic review of reviews

**DOI:** 10.1007/s10389-022-01753-9

**Published:** 2022-09-19

**Authors:** Aysegul Humeyra Kafadar, Gamze Gizem Tekeli, Katy A. Jones, Blossom Stephan, Tom Dening

**Affiliations:** 1 Academic Unit of Mental Health and Clinical Neuroscience, School of Medicine, University of Nottingham, Nottingham, UK; 2 Institute of Mental Health, Triumph Road, Nottingham, NG7 2TU UK

**Keywords:** Systematic review, SARS-CoV-2, COVID-19, Vaccination determinants, Vaccine hesitancy

## Abstract

**Aim:**

Although multiple COVID-19 vaccines are approved for global use, vaccine hesitancy poses a substantial risk for global health. Therefore, the aim of this umbrella review is to identify those factors that influence COVID-19 vaccination hesitancy in the general population. This is necessary to improve the effectiveness of future vaccination programmes.

**Methods:**

PubMed, Embase, Scopus, PsycInfo, the Cochrane Database of Systematic Reviews, Epistemonikos, and PROSPERO (International Prospective Register of Systematic Reviews) were searched on December 21, 2021. This review included reviews which investigated factors of intention, willingness, or hesitancy with regard to the COVID-19 vaccination in adult populations, with no restrictions on setting. Content-based structure was used to synthesise the extracted data. The findings were presented based on the Strategic Advisory Group of Experts (SAGE) Working Group Model for vaccine hesitancy.

**Results:**

A total of 3,392 studies were identified, of which 31 met the inclusion criteria. The most frequently documented factors associated with COVID-19 vaccine hesitancy included contextual factors, such as sex, age, and social inequalities; individual and group factors, such as trust in the healthcare system, public health authorities, and governments, and history of vaccination; vaccine-specific factors, such as concern for vaccine safety, perceived vaccine barriers, perceived effectiveness of vaccines, and concern about the rapid development of the vaccine; and disease-specific factors, such as fear of being infected with COVID-19, perceived severity of COVID-19, and knowledge of COVID-19.

**Conclusion:**

There are multiple factors associated with COVID-19 vaccine hesitancy. Our findings lay the foundation to further understand COVID-19 vaccination uptake and provide possible targets for intervention programmes. However, there are gaps in research concerning certain populations, including vaccination in people with mental disorders.

**Supplementary Information:**

The online version contains supplementary material available at 10.1007/s10389-022-01753-9.

## Introduction

COVID-19 is caused by the severe acute respiratory syndrome coronavirus 2 (SARS-CoV-2) (Holt et al. 2020) and reached the status of a pandemic on March 11, 2020 (Murphy et al. 2021). Globally, the pandemic has direct impact on social, economic, health, and healthcare systems (Brown et al. 2020; Kaye et al. 2021). COVID-19 encompasses a broad clinical spectrum that ranges from mild, self-limited illnesses to life-threatening, multiple organ involvement (Holt et al. 2020). While initially the main symptoms were considered to be pneumonia, respiratory symptoms, and in some cases, acute respiratory distress syndrome (ARDS) and shock (Fried et al. 2020), it is now known to also influence lungs, heart, and other organs, as well as blood vessels and central nervous system with a systemic inflammation (Hatmi 2021).

After COVID-19 was determined as a communicable disease, health professionals and governments launched new health measures to protect the public, including wearing face masks, social distancing, hand hygiene, and local and national lockdowns (Garg et al. 2021b; Low et al. 2021; Sharma 2021; WHO 2022). As with previous communicable diseases, vaccination is one of the most effective and cost-effective public health interventions (Al-Hanawi et al. 2021; Ehreth 2003; Eitze et al. 2021; Giubilini 2021; Paterson et al. 2016; Rémy et al. 2015; Wheelock et al. 2014). Hence, multiple SARS-CoV-2 vaccines have been generated and approved for global use, including Pfizer/BioNTech, Oxford/AstraZeneca, Moderna, Janssen, Sinopharm, and Sinovac-CoronaVac (Barda et al. 2021; Garg et al. 2021b; Livingston et al. 2021; Yan et al. 2021). To provide herd immunity, it was estimated at least 80% of the global population must be vaccinated (Iboi et al. 2020). There have been several studies in various countries and populations designed to determine individuals’ willingness to take up the COVID-19 vaccine. In their systematic review, Wang et al. (2021a) found the overall acceptance rate of the COVID-19 vaccination was 64.1% globally. Individuals with presence of chronic disease were found to have the highest acceptance rate (69.3%), while pregnant or breastfeeding women had the lowest (56.5%). Vaccination willingness rate has been found to range from 19.9% to 92.1% across different countries (Wang et al. 2021a).

Historically there has always been scepticism, fear, and refusal of vaccinations (Riedel 2005). Although the rate of COVID-19 vaccine intake has increased, the issue of vaccine hesitation has not been resolved (Al-Amer et al. 2021; Hajure et al. 2021; Our World in Data 2022; Wang et al. 2021a). This is because vaccine hesitancy has been defined on a continuum, from passive acceptance to active demand, by the Strategic Advisory Group of Experts (SAGE) Working Group on Vaccine Hesitancy (SAGE Working Group on Vaccine Hesitancy 2014). In other words, vaccine-hesitant people may accept vaccines while remaining unsure about them, some may delay or refuse some vaccines but accept others, and some people may refuse all vaccines (SAGE Working Group on Vaccine Hesitancy 2014). Vaccine hesitancy has risen over the decades (Wang et al. 2021a) because of safety and usefulness concerns (Dubé et al. 2013), and was one of the top ten global health issues in 2019 (WHO 2019). Increased vaccine hesitancy is often explained by the fact that social media accelerates the spread of non-evidence-based information and conspiracy theories (Mo et al. 2021). Vaccine hesitancy is a complex concept, and a wide range of factors has been suggested to explain it (SAGE Working Group on Vaccine Hesitancy 2014).

Given the overall burden of COVID-19 and the barriers to vaccination, efforts to mitigate vaccine hesitancy are reflected in the World Health Organization (WHO) priority of increasing vaccine acceptance. To address this initiative, there must be comprehensive understanding of the factors associated with COVID-19 vaccine hesitancy and of those groups who may be more likely to decline vaccination. This is challenging given the vast number of scientific articles investigating factors associated with COVID-19 vaccine hesitancy in different global populations over the last two years. As individual articles are difficult to report succinctly, many have been summarised in reviews. However, not all studies have used a specific theoretical framework to present their findings, which has made it difficult to classify and address factors of vaccine hesitation. It is therefore timely to synthesise all evidence about factors associated with vaccine hesitancy that we have to date.

The aim of this umbrella review is first to define factors of COVID-19 vaccine hesitancy amongst people aged 18 years or above in the general worldwide population, second to identify determinants of decision-making on COVID-19 vaccine acceptance, and third to use the SAGE theoretical framework to collate the evidence about facilitators and barriers of COVID-19 vaccine acceptance.

## Methods

### Data sources and searches

The Preferred Reporting Items for Systematic Reviews and Meta-Analyses (PRISMA–2009) was used to complete the review (Moher et al. 2009; Page et al. 2021). The protocol was registered on PROSPERO (CRD42021290887). A comprehensive search was performed in PubMed, Embase, Scopus, PsycInfo, the Cochrane Database of Systematic Reviews, Epistemonikos, and PROSPERO databases on December 21, 2021. Results were restricted to reviews published from the end of 2019 to December 21, 2021, in English. All quantitative and qualitative reviews (e.g., scoping, systematic, meta-analyses, and rapid reviews) evaluating COVID-19 vaccine acceptance, hesitancy or rejection, and underlying determinants associated with vaccine hesitancy were eligible.

The search keywords were tailored to each database and included the following: coronavirus terms (COVID-19 OR SARS-CoV-2 OR severe acute respiratory syndrome coronavirus 2 OR 2019-nCoV OR new coronavirus*) AND vaccine terms (vaccin* OR immunis* OR immuniz*) AND attitude and behavioural terms (anxi* OR attitude* OR awareness OR behavio?r* OR belief* OR critic* OR accept* OR confidence OR doubt* OR distrust OR dropout* OR exemption* OR fear* OR hesitan* OR concern* OR decision-making OR trust OR mistrust OR perception* OR refus* OR rejection OR rumo?r* OR compulsory OR anti-vaccin* OR anti-vaxx* OR intent* OR controvers* OR misconception* OR misinformation OR opposition OR delay OR knowledge OR dilemma* OR objector* OR objection* OR uptake OR barrier* OR enable* OR choice* OR mandatory). Backward citation searching was undertaken on all included reviews to ensure no reviews had been missed from the search.

### Study selection and data extraction

The electronic search results were exported to Rayyan software, and duplicates were auto-removed. Studies were evaluated for inclusion by the primary reviewer. Data of full-text reviews were extracted by one reviewer, with another independently evaluating 20% of these papers to determine eligibility and check for consistency. Any disagreements regarding study inclusion were resolved in consultation with a third reviewer. Information from the eligible studies was extracted using a pre-designed form in Excel including author, year of publication, study design, country, data collection period, number of included studies, population of interest, factors associated with intention to take COVID-19 vaccine, and reasons for vaccine hesitancy.

### Risk of bias assessment

The Joanna Briggs Institute (JBI) Critical Appraisal Tool for Systematic Reviews and Research Syntheses was used by two reviewers to assess the quality of the studies as low, moderate, or high. The JBI tool includes 11 assessment domains, e.g., well-articulated review question, inclusion criteria, search strategy, resource adequacy, critical appraisal tool, minimized risk of bias, data extraction, data synthesis method, publication bias, recommendation for policy and/or practice, and suggestions for future research (Aromataris et al. 2020). An 11-point scale was used for the umbrella review with a score of ≤ 4 indicating low quality, a score of 5–8 indicating moderate quality, and a score of 9–11 indicating high quality.

### Synthesis of results

Characteristics of selected studies and determinants of COVID-19 vaccine hesitancy are presented in tables. Results were summarised using content-based structure. A summary of the key findings on the determinants of COVID-19 vaccination intention and hesitancy is presented based on the framework developed by the SAGE Working Group on Vaccine Hesitancy (SAGE Working Group on Vaccine Hesitancy 2014). The model includes three domains for contextual, individual and group, and vaccine-specific determinants (SAGE Working Group on Vaccine Hesitancy 2014). Due to the current unexpected COVID-19 pandemic, a fourth domain was added regarding disease-specific determinants (Soares et al. 2021). A framework diagram is presented to summarise the most studied factors of COVID-19 vaccine hesitancy amongst the general worldwide population.

## Results

### Identification and selection of studies

**Fig. 1:**
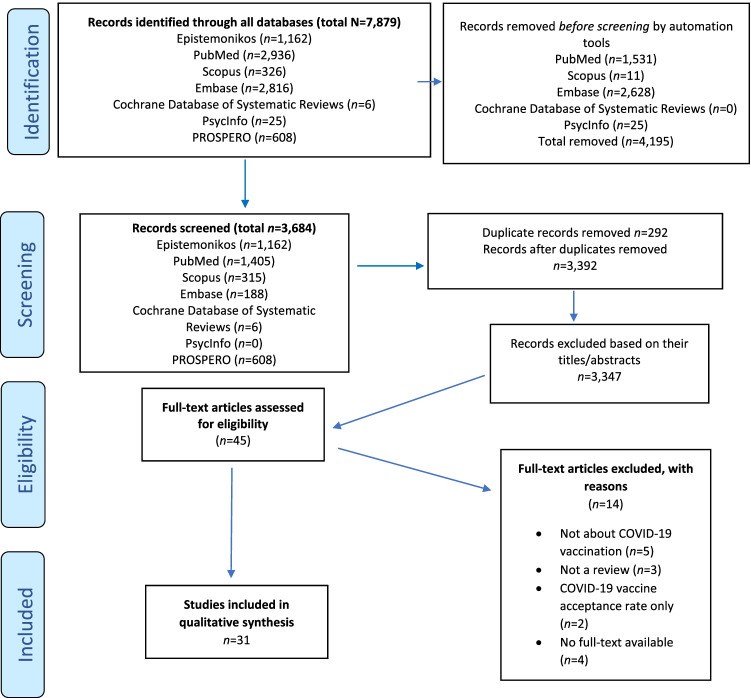
Searches according to PRISMA

In total, 7,879 studies were identified through the electronic search. After applying restrictions based on language, year, and full-text availability, 3,684 studies were retrieved from the seven databases. In total, *n* = 292 duplicates were removed via Rayyan (2022), leaving 3,392 studies for screening. Based on title and abstract screening 3,347 papers were excluded, and 45 full-text articles evaluated for eligibility. In total, 31 reviews met the eligibility criteria and were finally included (see Fig. 1).

### Features of the included studies

The main features of the 31 included studies are presented in Supplementary Table 1. They comprised systematic reviews (*n* =18), scoping reviews (*n* = 8), rapid reviews (*n* = 4), and a living review (*n* = 1). The studies were conducted in the general population (*n* = 20), healthcare workers (*n* = 5), minority ethnicity groups (*n* = 2), pregnant women who had at least primary education (*n* = 1), LGBTQ+ population (*n* = 1), Hispanics and African-Americans living in the United States (US) (*n* = 1), and older people (*n* = 1). Based on the JBI tool, 13 studies were rated as high quality, 18 as moderate quality, and none as low quality (see Supplementary Table 2). 

### Determinants of COVID-19 vaccine hesitancy

As shown in Table 1, the determinants of COVID-19 vaccine-related hesitancy were grouped into four main domains. Three of those, namely contextual factors, individuals and group factors, and vaccine-specific factors, were proposed by the SAGE Working Group Model on Vaccine Hesitancy, while the fourth domain was added by us to enable the exploration of disease-specific factors.

**Table 1:** Framework of SAGE Working Group Model on Vaccine Hesitancy (SAGE Working Group on Vaccine Hesitancy 2014), with the added fourth domain for COVID-19 disease-specific factors

Vaccine hesitancy associated factors	Predictors of vaccine hesitancy	Predictors of vaccine acceptance
Contextual factors		
Age (younger participants)	Aboelsaad et al. 2021; Ackah et al. 2021; Al-Amer et al. 2021; Al-Jayyousi et al. 2021; AlShurman et al. 2021; Aw et al. 2021; Biswas et al. 2021a; Cascini et al. 2021; Galanis et al. 2021a, b; Hajure et al. 2021; Januszek et al. 2021; Joshi et al. 2021; Khubchandani and Macias 2021; Li et al. 2021; Lin et al. 2020; Luo et al. 2021; Nehal et al. 2021; Ochieng et al. 2021; Robinson et al. 2021; Terry et al. 2021; Wake 2021; Wang et al. 2021b; Yasmin et al. 2021	
Sex (women)	Aboelsaad et al. 2021; Ackah et al. 2021; Al-Amer et al. 2021; Al-Jayyousi et al. 2021; AlShurman et al. 2021; Aw et al. 2021; Biswas et al. 2021a; Cascini et al. 2021; Galanis et al. 2021a, b; Hajure et al. 2021; Joshi et al. 2021; Khubchandani and Macias 2021; Li et al. 2021; Lin et al. 2020; Luo et al. 2021; Moola et al. 2021; Nehal et al. 2021; Ochieng et al. 2021; Robinson et al. 2021; Terry et al. 2021; Wake 2021; Wang et al. 2021b; Yasmin et al. 2021; Zintel et al. 2021	
*Social inequalities*		
Being an immigrant (not native-born)	Al-Jayyousi et al. 2021; Cascini et al. 2021; Galanis et al. 2021b; Ochieng et al. 2021	
Ethnicity (non-white)	Aboelsaad et al. 2021; Al-Jayyousi et al. 2021; AlShurman et al. 2021; Aw et al. 2021; Biswas et al. 2021a; Cascini et al. 2021; Galanis et al. 2021a, b; Garg et al. 2021a; Hajure et al. 2021; Joshi et al. 2021; Kamal et al. 2021; Li et al. 2021; Lin et al. 2020; Ochieng et al. 2021; Robinson et al. 2021; Terry et al. 2021; Veronese et al. 2021; Wake 2021; Wang et al. 2021b; Yasmin et al. 2021	
Having health insurance		Al-Jayyousi et al. 2021; AlShurman et al. 2021; Cascini et al. 2021; Joshi et al. 2021; Lin et al. 2020; Wake 2021
Larger household size	Khubchandani and Macias 2021; Lin et al. 2020	
Living in a rural area	AlShurman et al. 2021; Biswas et al. 2021a; Cascini et al. 2021; Galanis et al. 2021a; Joshi et al. 2021; Lin et al. 2020; Moola et al. 2021	
Living with others		Cascini et al. 2021; b; Li et al. 2021
Lower education level	Aboelsaad et al. 2021; Ackah et al. 2021; Al-Amer et al. 2021; Al-Jayyousi et al. 2021; AlShurman et al. 2021; Aw et al. 2021; Biswas et al. 2021a; Cascini et al. 2021; Galanis et al. 2021a, b; Hajure et al. 2021; Januszek et al. 2021; Joshi et al. 2021; Khubchandani and Macias 2021; Li et al. 2021; Moola et al. 2021; Ochieng et al. 2021; Robinson et al. 2021; Terry et al. 2021; Veronese et al. 2021; Wake 2021; Wang et al. 2021b; Yasmin et al. 2021	
Lower income	Aboelsaad et al. 2021; Ackah et al. 2021; Al-Jayyousi et al. 2021; AlShurman et al. 2021; Aw et al. 2021; Biswas et al. 2021a; Cascini et al. 2021; Galanis et al. 2021a, b; Hajure et al. 2021; Januszek et al. 2021; Joshi et al. 2021; Khubchandani and Macias 2021; Li et al. 2021; Lin et al. 2020; Moola et al. 2021; Ochieng et al. 2021; Robinson et al. 2021; Terry et al. 2021; Veronese et al. 2021; Wang et al. 2021b; Yasmin et al. 2021	
Marital status (married)	AlShurman et al. 2021; Moola et al. 2021	Aboelsaad et al. 2021; Al-Jayyousi et al. 2021
Occupation (working in the healthcare field/being healthcare workers)		Ackah et al. 2021; Al-Amer et al. 2021; AlShurman et al. 2021; Biswas et al. 2021a; Galanis et al. 2021a, b; Hajure et al. 2021; Joshi et al. 2021; Li et al. 2021; Moola et al. 2021
Presence of child/children	Aboelsaad et al. 2021; AlShurman et al. 2021	Ochieng et al. 2021; Wang et al. 2021b
Working in the private sector		Aboelsaad et al. 2021; AlShurman et al. 2021; Joshi et al. 2021; Wake 2021
*Pregnancy*		
Breastfeeding	Galanis et al. 2021b; Januszek et al. 2021; Yasmin et al. 2021	
Pregnancy	Galanis et al. 2021b; Januszek et al. 2021; Yasmin et al. 2021	
*Policies/politics*		
Believing in mandatory COVID-19 vaccination	Kamal et al. 2021	AlShurman et al. 2021; Hajure et al. 2021; Wake 2021
Certain political preferences/identities/ political leanings (liberals)		Al-Jayyousi et al. 2021; AlShurman et al. 2021; Aw et al. 2021; Crawshaw et al. 2021; Joshi et al. 2021; Li et al. 2021; Ochieng et al. 2021; Wake 2021; Wang et al. 2021b
Religious conviction	Al-Jayyousi et al. 2021; AlShurman et al. 2021; Biswas et al. 2021b; Joshi et al. 2021; Moola et al. 2021; Ochieng et al. 2021; Yasmin et al. 2021	
Individual and group factors		
*Trust and personal experiences*		
Belief that the authorities are motivated by financial gain rather than the health of people	Cascini et al. 2021; Januszek et al. 2021; Ochieng et al. 2021	
Confidence in vaccine developers		Aboelsaad et al. 2021; Ackah et al. 2021; Al-Amer et al. 2021; Al-Jayyousi et al. 2021; AlShurman et al. 2021; Aw et al. 2021; Biswas et al. 2021b; Cascini et al. 2021; Galanis et al. 2021a; Januszek et al. 2021; Kamal et al. 2021; Li et al. 2021; Moola et al. 2021; Ochieng et al. 2021; Terry et al. 2021; Wake 2021
History of racial discrimination	Khubchandani and Macias 2021	
Inconsistent risk message from public health organization	Biswas et al. 2021b; Li et al. 2021	
Lack of advocacy for vaccination by physicians	Aw et al. 2021	
Negative experiences of vaccines among family members/friends	Al-Jayyousi et al. 2021; Biswas et al. 2021b; Crawshaw et al. 2021; Galanis et al. 2021b; Lin et al. 2020; Moola et al. 2021; Ochieng et al. 2021	
Previous negative experiences with healthcare providers	Garg et al. 2021a	
Relying on CDC website for COVID-19 updates		AlShurman et al. 2021; Ochieng et al. 2021
Trust in government		Al-Amer et al. 2021; Al-Jayyousi et al. 2021; AlShurman et al. 2021; Aw et al. 2021; Biswas et al. 2021b; Cascini et al. 2021; Crawshaw et al. 2021; Hajure et al. 2021; Januszek et al. 2021; Joshi et al. 2021; Kamal et al. 2021; Li et al. 2021; Moola et al. 2021; Ochieng et al. 2021; Terry et al. 2021; Wake 2021; Wang et al. 2021b
Trust in healthcare system		Aboelsaad et al. 2021; Al-Amer et al. 2021; Al-Jayyousi et al. 2021; AlShurman et al. 2021; Aw et al. 2021; Biswas et al. 2021b; Cascini et al. 2021; Garg et al. 2021a; Hajure et al. 2021; Januszek et al. 2021; Joshi et al. 2021; Kamal et al. 2021; Khubchandani and Macias 2021; Li et al. 2021; Moola et al. 2021; Nehal et al. 2021; Ochieng et al. 2021; Terry et al. 2021; Wake 2021; Yasmin et al. 2021
Trust in public health authorities		Al-Amer et al. 2021; Al-Jayyousi et al. 2021; AlShurman et al. 2021; Aw et al. 2021; Biswas et al. 2021b; Cascini et al. 2021; Crawshaw et al. 2021; Hajure et al. 2021; Januszek et al. 2021; Joshi et al. 2021; Kamal et al. 2021; Khubchandani and Macias 2021; Li et al. 2021; Moola et al. 2021; Ochieng et al. 2021; Terry et al. 2021; Wake 2021; Yasmin et al. 2021
Trust in reputable information sources		Crawshaw et al. 2021
Unreliable messages on the approaches used for COVID-19 testing or testing delays	Ochieng et al. 2021	
*History of vaccination*		
Previously received an influenza vaccine		Aboelsaad et al. 2021; Al-Amer et al. 2021; Al-Jayyousi et al. 2021; AlShurman et al. 2021; Aw et al. 2021; Biswas et al. 2021a, b; Crawshaw et al. 2021; Galanis et al. 2021a; Hajure et al. 2021; Januszek et al. 2021; Joshi et al. 2021; Li et al. 2021; Lin et al. 2020; Luo et al. 2021; Ochieng et al. 2021; Terry et al. 2021; Wake 2021; Wang et al. 2021b
Receiving any vaccine in the past 5 years		Al-Jayyousi et al. 2021; AlShurman et al. 2021; Galanis et al. 2021a; Hajure et al. 2021; Januszek et al. 2021; Joshi et al. 2021; Lin et al. 2020; Ochieng et al. 2021; Wake 2021
*Beliefs and attitudes about health and prevention*		
Anti-vaccine movement (having a general anti-vaccine stance)	AlShurman et al. 2021; Biswas et al. 2021b; Joshi et al. 2021; Lin et al. 2020; Terry et al. 2021	
Belief in conspiracy theories	Al-Jayyousi et al. 2021; AlShurman et al. 2021; Biswas et al. 2021b; Cascini et al. 2021; Joshi et al. 2021; Ochieng et al. 2021	
Belief that exposure to infections gives the safest protection	AlShurman et al. 2021; Biswas et al. 2021b; Cascini et al. 2021; Li et al. 2021; Lin et al. 2020; Ochieng et al. 2021	
Belief that only people who are at risk of serious illness should be vaccinated	AlShurman et al. 2021; Wake 2021	
Belief that vaccination relieves worry about COVID-19		Al-Jayyousi et al. 2021; Kamal et al. 2021; Ochieng et al. 2021; Terry et al. 2021; Wake 2021
Believing rumours of infertility	Galanis et al. 2021b; Nehal et al. 2021; Ochieng et al. 2021	
Complacency	AlShurman et al. 2021; Aw et al. 2021; Biswas et al. 2021b; Cascini et al. 2021; Galanis et al. 2021a, b; Li et al. 2021; Wake 2021	
Compliance with community mitigation strategies		Biswas et al. 2021a; Cascini et al. 2021; Januszek et al. 2021; Kamal et al. 2021; Li et al. 2021; Ochieng et al. 2021; Wake 2021
Not believing COVID-19 virus was not developed in a laboratory		AlShurman et al. 2021; Joshi et al. 2021; Wake 2021
Trust in natural remedies	Biswas et al. 2021b; Joshi et al. 2021	
Trust in own immune system	Aboelsaad et al. 2021; AlShurman et al. 2021; Biswas et al. 2021b; Li et al. 2021	
Perception that disease can be prevented by vaccine/belief that COVID-19 vaccines help protect family		Aboelsaad et al. 2021; Al-Jayyousi et al. 2021; Aw et al. 2021; Cascini et al. 2021; Crawshaw et al. 2021; Galanis et al. 2021a, b; Januszek et al. 2021; Kamal et al. 2021; Moola et al. 2021; Ochieng et al. 2021; Terry et al. 2021; Wake 2021
*Altruistic traits*		
Individual or member of their household belonging to a vulnerable group		AlShurman et al. 2021; Cascini et al. 2021; Lin et al. 2020; Wake 2021
Self-efficacy		Biswas et al. 2021a
Viewing COVID-19 vaccination as a social/collective responsibility		AlShurman et al. 2021; Biswas et al. 2021a; Cascini et al. 2021; Crawshaw et al. 2021; Galanis et al. 2021b; Hajure et al. 2021; Januszek et al. 2021; Li et al. 2021; Ochieng et al. 2021; Wake 2021
Willingness to protect others by getting oneself vaccinated		AlShurman et al. 2021; Biswas et al. 2021a; Cascini et al. 2021; Hajure et al. 2021; Januszek et al. 2021; Kamal et al. 2021; Li et al. 2021; Lin et al. 2020; Moola et al. 2021; Ochieng et al. 2021; Wake 2021; Wang et al. 2021b
*Other*		
Presence of chronic disease/self-reported health outcomes	Ackah et al. 2021	Aboelsaad et al. 2021; Al-Jayyousi et al. 2021; AlShurman et al. 2021; Biswas et al. 2021a; Galanis et al. 2021a, b; Hajure et al. 2021; Joshi et al. 2021; Li et al. 2021; Wake 2021; Wang et al. 2021b
Psychological distress symptoms (stress, depression, anxiety)		Al-Jayyousi et al. 2021; Crawshaw et al. 2021; Hajure et al. 2021; Li et al. 2021
Social concern regarding COVID-19 vaccine stigma	Garg et al. 2021a	
Use of social media for COVID-19 vaccine-related information	Ackah et al. 2021; Al-Amer et al. 2021; Al-Jayyousi et al. 2021; AlShurman et al. 2021; Aw et al. 2021; Biswas et al. 2021b; Kamal et al. 2021; Moola et al. 2021; Ochieng et al. 2021	Cascini et al. 2021; Hajure et al. 2021
Vaccine-specific factors		
*COVID-19 vaccine safety and effectiveness*		
Concerns about COVID-19 vaccine necessity	AlShurman et al. 2021; Cascini et al. 2021; Crawshaw et al. 2021; Lin et al. 2020	
Concerns about rapid development of vaccine and/or its mechanism of action/gaps in knowledge about COVID-19 vaccines	Al-Jayyousi et al. 2021; AlShurman et al. 2021; Aw et al. 2021; Biswas et al. 2021b; Cascini et al. 2021; Crawshaw et al. 2021; Joshi et al. 2021; Li et al. 2021; Moola et al. 2021; Ochieng et al. 2021; Terry et al. 2021	
Development of vaccine in non-first world country	AlShurman et al. 2021; Aw et al. 2021; Joshi et al. 2021; Lin et al. 2020; Ochieng et al. 2021	
Having COVID-19 vaccine safety concern	Aboelsaad et al. 2021; Ackah et al. 2021; Al-Amer et al. 2021; Al-Jayyousi et al. 2021; AlShurman et al. 2021; Aw et al. 2021; Biswas et al. 2021a, b; Cascini et al. 2021; Crawshaw et al. 2021; Galanis et al. 2021b; Garg et al. 2021a; Hajure et al. 2021; Januszek et al. 2021; Joshi et al. 2021; Kamal et al. 2021; Khubchandani and Macias 2021; Li et al. 2021; Lin et al. 2020; Moola et al. 2021; Nehal et al. 2021; Ochieng et al. 2021; Terry et al. 2021; Wake 2021; Wang et al. 2021b; Yasmin et al. 2021	
Longer duration of immunity		AlShurman et al. 2021; Joshi et al. 2021; Lin et al. 2020; Ochieng et al. 2021
Number of injections/doses (more doses)	AlShurman et al. 2021; Ochieng et al. 2021	
Perceived efficacy or effectiveness of COVID-19 vaccine		Aboelsaad et al. 2021; Al-Amer et al. 2021; Al-Jayyousi et al. 2021; AlShurman et al. 2021; Aw et al. 2021; Biswas et al. 2021a, b; Cascini et al. 2021; Crawshaw et al. 2021; Galanis et al. 2021b; Garg et al. 2021a; Hajure et al. 2021; Januszek et al. 2021; Joshi et al. 2021; Kamal et al. 2021; Khubchandani and Macias 2021; Li et al. 2021; Lin et al. 2020; Moola et al. 2021; Nehal et al. 2021; Ochieng et al. 2021; Terry et al. 2021; Wake 2021; Wang et al. 2021b; Yasmin et al. 2021
Perceived potential vaccine harms	Aboelsaad et al. 2021; Ackah et al. 2021; Al-Amer et al. 2021; Al-Jayyousi et al. 2021; Aw et al. 2021; Biswas et al. 2021b; Cascini et al. 2021; Crawshaw et al. 2021; Hajure et al. 2021; Januszek et al. 2021; Joshi et al. 2021; Kamal et al. 2021; Khubchandani and Macias 2021; Li et al. 2021; Lin et al. 2020; Moola et al. 2021; Nehal et al. 2021; Ochieng et al. 2021; Terry et al. 2021; Wake 2021; Wang et al. 2021b; Yasmin et al. 2021	
*Knowledge about COVID-19 vaccine*		
Better informed about COVID-19 vaccines		Galanis et al. 2021b; Wake 2021
Knowing higher number of people vaccinated		Lin et al. 2020; Ochieng et al. 2021
*Vaccine beliefs and attitudes*		
Belief that vaccines can stop the pandemic		Aboelsaad et al. 2021; Wake 2021
Positive attitudes/perceived benefit of vaccine		Aboelsaad et al. 2021; Ackah et al. 2021; Al-Amer et al. 2021; Al-Jayyousi et al. 2021; AlShurman et al. 2021; Aw et al. 2021; Biswas et al. 2021a, b; Cascini et al. 2021; Crawshaw et al. 2021; Galanis et al. 2021a; Hajure et al. 2021; Januszek et al. 2021; Joshi et al. 2021; Kamal et al. 2021; Khubchandani and Macias 2021; Li et al. 2021; Lin et al. 2020; Moola et al. 2021; Nehal et al. 2021; Ochieng et al. 2021; Terry et al. 2021; Wake 2021; Wang et al. 2021b
*Perceived barriers to vaccination*		
Access issues in terms of convenience, time, and cost	AlShurman et al. 2021; Biswas et al. 2021b; Cascini et al. 2021; Crawshaw et al. 2021; Joshi et al. 2021; Kamal et al. 2021; Lin et al. 2020; Moola et al. 2021; Ochieng et al. 2021; Wake 2021	
Perceived vaccine barriers	Ackah et al. 2021; Al-Amer et al. 2021; Al-Jayyousi et al. 2021; AlShurman et al. 2021; Aw et al. 2021; Biswas et al. 2021b; Cascini et al. 2021; Hajure et al. 2021; Joshi et al. 2021; Kamal et al. 2021; Lin et al. 2020; Moola et al. 2021; Nehal et al. 2021; Ochieng et al. 2021; Wake 2021; Wang et al. 2021b	
Vaccine recommendation from CDC, FDA, WHO, or healthcare professionals		Al-Jayyousi et al. 2021; AlShurman et al. 2021; Crawshaw et al. 2021; Hajure et al. 2021; Joshi et al. 2021; Lin et al. 2020; Moola et al. 2021; Ochieng et al. 2021; Wake 2021
Disease-specific factors		
*Knowledge*		
Knowledge about COVID-19		Ackah et al. 2021; Al-Amer et al. 2021; Al-Jayyousi et al. 2021; AlShurman et al. 2021; Biswas et al. 2021a, b; Cascini et al. 2021; Galanis et al. 2021b; Januszek et al. 2021; Joshi et al. 2021; Lin et al. 2020; Moola et al. 2021; Ochieng et al. 2021; Wake 2021; Wang et al. 2021b
*Perceptions about COVID-19*		
Belief that COVID-19 is highly contagious and lethal		Al-Jayyousi et al. 2021; Wake 2021
Perception that COVID-19 will persist/ belief that next COVID-19 waves are coming		AlShurman et al. 2021; Joshi et al. 2021; Wake 2021
*Perceived risk and severity*		
Belief that lockdown periods decrease the number of cases	Biswas et al. 2021b	
Encounters with suspected or confirmed COVID-19 patients		Ackah et al. 2021; AlShurman et al. 2021; Biswas et al. 2021a; Galanis et al. 2021a; Joshi et al. 2021; Kamal et al. 2021; Li et al. 2021; Wake 2021
Fear about being infected with COVID-19 and its impact		Aboelsaad et al. 2021; Ackah et al. 2021; Al-Amer et al. 2021; Al-Jayyousi et al. 2021; AlShurman et al. 2021; Aw et al. 2021; Biswas et al. 2021a, b; Cascini et al. 2021; Crawshaw et al. 2021; Galanis et al. 2021a; Hajure et al. 2021; Januszek et al. 2021; Joshi et al. 2021; Kamal et al. 2021; Li et al. 2021; Lin et al. 2020; Moola et al. 2021; Ochieng et al. 2021; Terry et al. 2021; Wake 2021; Wang et al. 2021b
Member(s) of family/close social network infected with COVID-19		Crawshaw et al. 2021
Perceived risk of COVID-19	Khubchandani and Macias 2021	Aboelsaad et al. 2021; Ackah et al. 2021; Al-Amer et al. 2021; Al-Jayyousi et al. 2021; AlShurman et al. 2021; Aw et al. 2021; Biswas et al. 2021a, b; Cascini et al. 2021; Crawshaw et al. 2021; Galanis et al. 2021a, b; Hajure et al. 2021; Januszek et al. 2021; Joshi et al. 2021; Kamal et al. 2021; Li et al. 2021; Lin et al. 2020; Moola et al. 2021; Ochieng et al. 2021; Terry et al. 2021; Wake 2021; Wang et al. 2021b
Perceived severity of COVID-19		Aboelsaad et al. 2021; Al-Amer et al. 2021; Al-Jayyousi et al. 2021; AlShurman et al. 2021; Aw et al. 2021; Biswas et al. 2021a, b; Cascini et al. 2021; Galanis et al. 2021a, b; Hajure et al. 2021; Januszek et al. 2021; Joshi et al. 2021; Khubchandani and Macias 2021; Li et al. 2021; Lin et al. 2020; Moola et al. 2021; Ochieng et al. 2021; Terry et al. 2021; Wake 2021; Wang et al. 2021b
Prior COVID-19 infection/being tested positive for COVID-19 in the past	Biswas et al. 2021a; Galanis et al. 2021b; Hajure et al. 2021	Ackah et al. 2021; AlShurman et al. 2021; Kamal et al. 2021; Lin et al. 2020; Wake 2021
Taking direct care of COVID-19 patients		Biswas et al. 2021a; Kamal et al. 2021; Li et al. 2021; Wake 2021

*CDC*, Centers for Disease Control and Prevention; *FDA*, Food and Drug Administration; *WHO*, World Health Organization

### Context-related determinants

Contextual factors of COVID-19 vaccine hesitancy were categorized in six subgroups, namely age, sex, social inequalities, pregnancy, policies, and religious conviction. Female sex, being younger, and social inequalities, such as being of non-white ethnicity, living in a rural area, having a lower education and income level, and having a larger household size, being pregnant, and having a conservative religious conviction were factors that increase vaccine hesitancy. In contrast, some social inequalities such as occupation, for example working in the healthcare field or being a physician rather than nurse or paramedical staff (Ackah et al. 2021; Al-Amer et al. 2021; AlShurman et al. 2021; Biswas et al. 2021a; Galanis et al. 2021a; Hajure et al. 2021; Joshi et al. 2021; Li et al. 2021; Moola et al. 2021), living with others (Cascini et al. 2021; Galanis et al. 2021b; Li et al. 2021), working in the private sector (Aboelsaad et al. 2021; AlShurman et al. 2021; Joshi et al. 2021; Wake 2021), and having health insurance (Al-Jayyousi et al. 2021; AlShurman et al. 2021; Cascini et al. 2021; Joshi et al. 2021; Lin et al. 2020; Wake 2021), were found to increase the desire to be vaccinated. Political views were also associated with making vaccination decisions. Being liberal in political leaning (Al-Jayyousi et al. 2021; AlShurman et al. 2021; Aw et al. 2021; Crawshaw et al. 2021; Joshi et al. 2021; Li et al. 2021; Ochieng et al. 2021; Wake 2021; Wang et al. 2021b) and belief that COVID-19 vaccination is mandatory were found to increase vaccine uptake (AlShurman et al. 2021; Hajure et al. 2021; Wake 2021). However, a study among healthcare workers in the UK noted concerns from participants that vaccination mandates would cause ethnic and racial divides between communities and increase stigma and discrimination (Kamal et al. 2021). Furthermore, factors such as being married and having a child were found to show contradictory results. While two studies showed that being married had a positive effect on intention to get vaccinated (Aboelsaad et al. 2021; Al-Jayyousi et al. 2021), two other studies found a negative effect with vaccine hesitancy (AlShurman et al. 2021; Moola et al. 2021). Similarly, having a child was both a barrier to (Aboelsaad et al. 2021; AlShurman et al. 2021) and a facilitator of (Ochieng et al. 2021; Wang et al. 2021b) vaccine hesitancy.

### Individual and group-related determinants

Five subgroups were classed as individual and group determinants of COVID-19 vaccine hesitancy, namely trust and personal experiences, history of vaccination, beliefs and attitudes about health and prevention, altruistic traits, and others. Trust and personal experiences, such as having negative experiences of vaccines among family members or friends, receiving inconsistent and unreliable risk messages from public health organizations, and mistrust in public health authorities, the healthcare system, government, and vaccine developers were all associated with increased vaccine hesitancy. Certain beliefs and attitudes about health and prevention, such as conspiracy theories (Al-Jayyousi et al. 2021; AlShurman et al. 2021; Biswas et al. 2021b; Cascini et al. 2021; Joshi et al. 2021; Ochieng et al. 2021), believing rumours of infertility (Galanis et al. 2021b; Nehal et al. 2021; Ochieng et al. 2021), trust in natural remedies (Biswas et al. 2021b; Joshi et al. 2021) and one’s own immune system (Aboelsaad et al. 2021; AlShurman et al. 2021; Biswas et al. 2021b; Li et al. 2021), belief that natural exposure to infections gives the safest protection (AlShurman et al. 2021; Biswas et al. 2021b; Cascini et al. 2021; Li et al. 2021; Lin et al. 2020; Ochieng et al. 2021), and believing only individuals who are at risk of serious illness should be vaccinated (AlShurman et al. 2021; Wake 2021) were found to be associated with increased vaccine hesitancy. In contrast, other attitudes and behaviours, such as compliance with community mitigation strategies and belief that vaccination relieves worry about COVID-19, were associated with increased vaccination acceptance. Additionally, a history of vaccination and altruistic traits, such as willingness to protect others by getting vaccinated, individual or someone in own household belonging to a vulnerable group, and viewing COVID-19 vaccination as a collective responsibility were found to be facilitators to vaccination. Some reviews reported that having psychological distress symptoms was a facilitator of vaccination (Al-Jayyousi et al. 2021; Crawshaw et al. 2021; Hajure et al. 2021; Li et al. 2021). While presence of chronic disease has been reported to increase vaccine uptake in many studies (Aboelsaad et al. 2021; Al-Jayyousi et al. 2021; AlShurman et al. 2021; Biswas et al. 2021a; Galanis et al. 2021a, b; Hajure et al. 2021; Joshi et al. 2021; Li et al. 2021; Wake 2021; Wang et al. 2021b), in a scoping review conducted in Africa, the presence of chronic disease was found to be a predictor of vaccine hesitancy (Ackah et al. 2021). Using social media for COVID-19 vaccine-related information also affected willingness to get vaccinated both positively (Cascini et al. 2021; Hajure et al. 2021) and negatively (Ackah et al. 2021; Al-Amer et al. 2021; Al-Jayyousi et al. 2021; AlShurman et al. 2021; Aw et al. 2021; Biswas et al. 2021b; Kamal et al. 2021; Moola et al. 2021; Ochieng et al. 2021).

### Vaccine-specific determinants

Vaccine-specific determinants of COVID-19 vaccine hesitancy were of four types, namely concerns about COVID-19 vaccine safety and effectiveness, knowledge about the COVID-19 vaccines, beliefs and attitudes about the vaccines, and perceived barriers. Among these, the most documented determinants associated with enhanced hesitancy included having a COVID-19 vaccine safety and effectiveness concern, perceived barriers, concerns about the development process of vaccines, perceived potential vaccine harm, a higher number of doses (AlShurman et al. 2021; Ochieng et al. 2021), and COVID-19 vaccine necessity (AlShurman et al. 2021; Cascini et al. 2021; Crawshaw et al. 2021; Lin et al. 2020). Otherwise, positive attitudes toward vaccination, recommendation of the vaccine by the Centers for Disease Control and Prevention (CDC), WHO, healthcare professionals, scientists, social services, religious leaders, or an influential community member (Al-Jayyousi et al. 2021; AlShurman et al. 2021; Crawshaw et al. 2021; Hajure et al. 2021; Joshi et al. 2021; Lin et al. 2020; Moola et al. 2021; Ochieng et al. 2021; Wake 2021), longer duration of immunity (AlShurman et al. 2021; Joshi et al. 2021; Lin et al. 2020; Ochieng et al. 2021), a greater number of people vaccinated (Lin et al. 2020; Ochieng et al. 2021), more information about COVID-19 vaccines (Galanis et al. 2021b; Wake 2021), and believing vaccination can stop the pandemic (Aboelsaad et al. 2021; Wake 2021) were all associated with increased willingness to get vaccinated.

### Disease-specific determinants

Three subgroups were identified as being related to COVID-19 disease-related determinants of vaccine hesitancy, namely knowledge, perceptions about COVID-19, and perceived risk and severity of COVID-19. The most common determinants of COVID-19 vaccine acceptance were perceived risk of COVID-19, fear about being infected with COVID-19 and its impact (Aboelsaad et al. 2021; Ackah et al. 2021; Al-Amer et al. 2021; Al-Jayyousi et al. 2021; AlShurman et al. 2021; Aw et al. 2021; Biswas et al. 2021a, b; Cascini et al. 2021; Crawshaw et al. 2021; Hajure et al. 2021; Januszek et al. 2021; Joshi et al. 2021; Kamal et al. 2021; Li et al. 2021; Lin et al. 2020; Moola et al. 2021; Ochieng et al. 2021; Terry et al. 2021; Wake 2021; Wang et al. 2021b), perceived severity of COVID-19 (Aboelsaad et al. 2021; Al-Amer et al. 2021; Al-Jayyousi et al. 2021; AlShurman et al. 2021; Aw et al. 2021; Biswas et al. 2021a, b; Cascini et al. 2021; Galanis et al. 2021a, b; Hajure et al. 2021; Januszek et al. 2021; Joshi et al. 2021; Khubchandani and Macias 2021; Li et al. 2021; Lin et al. 2020; Moola et al. 2021; Ochieng et al. 2021; Terry et al. 2021; Wake 2021; Wang et al. 2021b), COVID-19 knowledge (Ackah et al. 2021; Al-Amer et al. 2021; Al-Jayyousi et al. 2021; AlShurman et al. 2021; Biswas et al. 2021a, b; Cascini et al. 2021; Galanis et al. 2021b; Januszek et al. 2021; Joshi et al. 2021; Lin et al. 2020; Moola et al. 2021; Ochieng et al. 2021; Wake 2021; Wang et al. 2021b), encountering suspected or confirmed patients with COVID-19, taking direct care of patients with COVID-19 (Biswas et al. 2021a; Kamal et al. 2021; Li et al. 2021; Wake 2021), perception that COVID-19 will persist (AlShurman et al. 2021; Joshi et al. 2021; Wake 2021), and believing COVID-19 is contagious and lethal (Al-Jayyousi et al. 2021; Wake 2021). Only one review reported that members of families or close social networks having been infected with COVID-19 increased the desire to get vaccinated (Crawshaw et al. 2021). Conversely, lockdown periods reducing the number of cases were associated with increased vaccine hesitancy (Biswas et al. 2021b). Prior COVID-19 infection was found to be both an enhancer (Biswas et al. 2021a; Galanis et al. 2021b; Hajure et al. 2021) and reducer (Ackah et al. 2021; AlShurman et al. 2021; Kamal et al. 2021; Lin et al. 2020; Wake 2021) of vaccine hesitancy.

### Vaccine hesitancy in specific populations

Contrary to the general population, pregnant women were hesitant to get vaccinated due to fear of harming the foetus. The explicit communication about COVID-19 vaccine safety increased willingness to take up the vaccine among pregnant women (Januszek et al. 2021). For minority ethnicities, increased visibility of less well-represented groups in the media and proactive engagement of healthcare professionals from diverse ethnic backgrounds was associated with increased vaccine acceptance (Kamal et al. 2021). In Muslim populations, the belief that the COVID-19 vaccine include non-Halal or alcohol-based ingredients, and concerns that the side-effects might impact Ramadan, increased vaccine hesitancy (Ochieng et al. 2021). In Africa, lack of support from employers, being discouraged by religious leaders, and presence of chronic disease were some of the reasons for vaccine hesitancy (Ackah et al. 2021). Amongst Hispanics and African-Americans in the US, the perceived risk of getting infected with COVID-19 was associated with increased vaccine hesitancy, unlike the general population, where it was associated with increased willingness to take the COVID-19 vaccine (Khubchandani and Macias 2021). In the LGBTQ+ population, social concern regarding COVID-19 vaccine stigma and previous negative experiences with healthcare providers were a factor for vaccine hesitancy (Garg et al. 2021a) (Supplementary Table 1).

## Discussion

To the best of our knowledge, this is the first umbrella review describing contextual, individual and group, vaccine-specific, and disease-specific hesitancy factors associated with the COVID-19 vaccination. The findings highlight that vaccine hesitancy is complex and associated with 79 factors in four categories with differing frequency and importance.

The most important contextual factors were sex, age, and some social inequalities such as ethnicity, lower education, and income level. Among the individual and group factors, the most frequently encountered factors were related to information sources, trust, and personal experiences. The most documented vaccine-specific determinants that impact individuals’ willingness to vaccinate were COVID-19 vaccine safety and effectiveness, perceived vaccine barriers, concern about the rapid development of the COVID-19 vaccine, and inadequate knowledge status for COVID-19 vaccines. Furthermore, disease-related factors affecting vaccine hesitancy were associated with knowledge and perception of COVID-19. Increasing knowledge, perceived risk of COVID-19 infection, and perceived COVID-19 severity were among the most prominent factors increasing vaccine acceptance.

Vaccine hesitancy is complex and associated with factors which are both non-modifiable, such as sex, age, or ethnicity, and modifiable. Some factors may be time limited. For example, those planning a family, or currently pregnant or breastfeeding, may be hesitant for specific reasons related to fertility and foetal health, with these concerns dissipating after this stage of life. Solving deep-rooted issues such as having a consistent anti-vaccine stance, previous negative experiences with vaccines or healthcare providers, having religious restrictions, a history of racial discrimination, or believing in conspiracy theories may require longer and more complex intervention.

To enhance COVID-19 vaccine uptake, health professionals should liaise with government authorities to improve vaccine education and awareness, provide resources for vaccination programs, organise local vaccination drives, and create ideas to solve access and cost issues related to COVID-19 vaccination. People with some non-modifiable risk factors, such as religious conservatism, and ethnic minorities may nonetheless be influenced towards vaccine acceptance by community leaders or targeted public health information. Therefore, governments, healthcare professionals, public health organizations, and media platforms should provide reliable and evidence-based information as well as vaccine distribution strategies, and health messages should focus on local communities and be available in multiple languages (SAGE Ethnicity Group 2020). Media literacy education, including news literacy, health media literacy, digital literacy, and media and information literacy, should be provided for all ages, with a suitable pedagogical approach for each age group (Rasi et al. 2019).

The review has several strengths. An extensive search of seven different databases was conducted. The reviews included were up-to-date and included data from many hundreds of individual published studies. Twenty per cent of full-text reviews were screened by two reviewers, and any disagreement was solved by an experienced reviewer. These methods were replicated for quality assessment. The study presented factors of vaccine hesitancy within the SAGE Working Group Model with an additional disease-specific factor category. To clarify reasons for vaccine hesitancy, the substantial determinants identified in this study were discussed.

However, there are also some limitations. First, the selected reviews included studies that were conducted before the launch of any COVID-19 vaccine. Therefore, individuals’ opinions might have changed over time, especially during emerging evidence and messages over different phases of the pandemic. It proved impossible to quantify exactly how many individual studies included in the reviews may have been published before vaccines were available. Second, only literature in English was included, which could have led to omissions or limit the generalisability of our findings. Third, grey literature was excluded, meaning that valuable sources of information could have been missed. Fourth, the review does not include the acceptance rate of vaccination for each included population. Finally, there were very few studies conducted to identify the determinants of vaccine hesitancy for vulnerable populations such as people with mental disorders, such as communication disorders, bipolar and related disorders, neurocognitive disorders, anxiety disorders, depressive disorders, sleep disorders, substance-related disorders, and schizophrenia, etc., indicating a significant gap in the literature for future research.

## Conclusion

Overall, COVID-19 vaccine hesitancy is a prevalent global issue. Women, and individuals who were younger, pregnant, or living with social inequalities were more likely to be hesitant. Increased vaccine uptake was associated with clear communication about COVID-19 vaccines, and enhanced levels of COVID-19 and COVID-19 vaccine knowledge. Improving trust and delivering consistent and reliable information seem to play a crucial role in reducing vaccine hesitancy. Healthcare providers need to be aware of these factors when formulating policies regarding COVID-19 vaccine and public health messages. Distinct populations, such as pregnant women or people with mental health problems, may benefit from more focused information specifically for their needs.

## Supplementary information

Table S1: characteristics of the selected studies in the umbrella review on determinants of vaccine hesitancy. 

Table S2: JBI checklist for systematic review and research syntheses.

ESM 1 (DOCX 77 kb)

ESM 2 (DOCX 65 kb)
