# Supplementary material for: Determinants for COVID-19 vaccine hesitancy in the general population: a systematic review of reviews
Source: Z Gesundh Wiss. 2022 Sep 19:1–17. Online ahead of print. doi: 10.1007/s10389-022-01753-9 (PMC9483252; doi:10.1007/s10389-022-01753-9)
Supplement: Supplementary file 1 — (DOCX 77 kb) [file 10389_2022_1753_MOESM1_ESM.docx]

**Title: Determinants for COVID-19 vaccine hesitancy in the general population: A systematic review of reviews**

***Journal of Public Health ISSN 1613-2238***

**Supplementary Table 1** **Characteristics of the selected studies into the umbrella review on determinants of vaccine hesitancy**

| **No** | **Author-Year** | **Study design** | **Number of studies in review (n)** | **Data collection period** | **Country** | **Population of interest** | **Factors associated with intention to take COVID-19 vaccine** | **Reason for vaccine hesitancy** |
| --- | --- | --- | --- | --- | --- | --- | --- | --- |
| 1 | Aboelsaad et al. 2021 | Systematic review and meta-analysis | 39 | Until end of January 2021 | Global | General population | Being male, older age, higher education, and income level, receiving influenza vaccine, professional private work, being married, presence of trusted health systems, fear of getting infected with virus, presence of chronic disease | Not being employed in a full-time job or retired, being single or widowed, Black race or mixed ethnicity, concerns about vaccine's efficacy and effectiveness, vaccine side effects, trust in own immune system |
| 2 | Ackah et al. 2021 | Scoping review | 16 | 1 January 2020–5 July 2021 | Africa | General population | Being male, higher level of education, working in a health-related occupation (being a physician), greater knowledge of COVID-19, fear of virus (including having flu-like symptoms, being tested for COVID-19, or relatives who contracted virus), being 16 to 30 years old, positive perceptions towards vaccine sources and pharmaceutical industry, higher income, and effective media communication | Concern about vaccine safety, poor vaccine promotion with conflicting information, misinformation from social media, fear of getting ill or side effects from vaccine, discouragement from some religious leaders, lack of belief in existence of COVID-19, belief in one's own immunity, lack of support from employers, presence of chronic disease |
| 3 | Al-Amer et al. 2021 | Systematic review | 30 | On or before 31 December 2020 | Global | General population | Being older, male, higher level education, previous influenza vaccination, perceiving oneself to be at greater risk of exposure or greater susceptibility to COVID-19, having high levels of trust in health-related information from government bodies and healthcare providers | High exposure to negative information about COVID-19 vaccines, particularly in social media, certain health professional’ groups including nurses and nursing assistants, concerns regarding vaccine safety and efficacy, mistrust of health authorities |
| 4 | Al-Jayyousi et al. 2021 | PRISMA scoping review | 50 | 1 January 2020–15 February 2021 | Global | General population | Being White, older than 25, higher education and income, male, presence of chronic disease, employed, married with children, having health insurance, belief that coronavirus is contagious and lethal, high information on health literacy, being stressed, worried, and anxious about getting infected, trusting healthcare system, government, and companies producing vaccines, positive experience with previous vaccines, recommendation by social services or healthcare providers, positive vaccination view of family or friends | Conspiracy beliefs, political conservatism, religious conviction, misleading information shared on social media platforms, negative perception towards vaccine efficacy, safety, and side effects or toxicity of vaccine, belief that immunity boosted by a vaccine would be for a short period only, and that the vaccines are most probably fake, concerns about rapid development of vaccine |
| 5 | AlShurman et al. 2021 | Scoping review-modified version of Arksey and O'Malley strategy | 48 | November 2019–December 2020 | Global | Adults aged 18–60 | Male sex, older age, higher education level, White ethnicity, working in a health-related occupation, liberal political views, prosocial behaviours and social contacts/activities, higher trust in scientist, CDC, healthcare professionals and state health department, taking recommendations from CDC/FDA/WHO, work stress and anxiety, trust in vaccines, being at very high risk of severe COVID-19, higher vaccine efficacy, previously received influenza vaccine, lower vaccine price, vaccine’s country of origin (US), fear of COVID-19, presence of chronic disease, immunocompromised individuals or family with compromised immune systems, private health insurance, being sick with COVID-19, recommendation by health professionals/scientist, high proportion of vaccinated acquaintances | Lower income, being unemployed or retired, living in rural settings, being married, having children, belief that media have over-exaggerated risk of COVID-19, timeline for outbreak will be short, child protection and parental concern, mistrust in government and pharmaceutical companies, concern for vaccine efficacy and safety, anti-vaccine attitudes, conspiracy beliefs, preference for natural immunity, believing COVID-19 vaccination is unnecessary, fear of adverse side effects from vaccine, concerns for rapid vaccine development, not having health insurance, financial barriers, being exposed to information about COVID-19 on social media |
| 6 | Aw et al. 2021 | PRISMA  scoping review | 97 | December 2019–March 2021 | High-income countries | General population, HCWs, university students/staffs, patients | Male sex, older age, ethnicity other than White, higher education, higher income level, political leaning (liberal), higher trust in government, pharmaceutical industry, science, and healthcare system, previously received influenza vaccine | Consumers of non-traditional sources of information or users of social media/internet, belief vaccines are not safe or efficacious, concerns about rapid development of vaccine and/or its mechanism of action, increased perceived barriers to accessibility of vaccine, lack of advocacy for vaccination by physicians, development of vaccine in non-first world country, lesser fear for health, low risk perception of contracting COVID-19, strong belief COVID-19 is not severe |
| 7 | N. Biswas et al. 2021 | Scoping review | 35 | February 2020–February 2021 | Global | HCWs | Being male, older age, doctoral or postgraduate degree, higher perceived risk of getting infected with COVID-19, fear of being infected with COVID-19, direct patient contact, caring for COVID patients, history of influenza vaccination, compliance with vaccines, confidence in vaccines, White or Asian race, higher income, medical risk and presence of chronic disease, not being infected with COVID-19 in the past, knowledge about COVID-19 infection and disease, working in non-rural areas, beliefs that vaccines may protect friends, family, and community members | Concerns about vaccine safety, efficacy, and potential side effects |
| 8 | R. Biswas et al. 2021 | PRISMA-scoping review | 82 | 19 February 2020–22 February 2021 | Global | General population | Believe in vaccine safety and efficacy, trust in healthcare system, government, and vaccine manufacturer | Vaccine side effects, individuals believe they are at less risk of getting to infected by COVID-19, religious beliefs, price of vaccine and lack of insurance, rapid development of vaccine, widespread misinformation in the social media, negative past vaccine experience, political instability, racial and ethnic minority, lockdown periods decreasing the number of cases, trust in natural remedies, lack of information about vaccine, inconsistent risk message from public health organizations, anti-vaccine movement |
| 9 | Cascini et al. 2021 | Systematic review | 209 | 24 February 2021–5 July 2021 | Global | General population | Having trust in medical professionals and government, expressing their worry about the outbreak, being more exposed to social media coverage, and showing a greater medical and scientific understanding of the virus, higher compliancy with proposed preventive measures for stopping spread of COVID-19 | Negative perception of vaccine efficacy, safety, convenience, and price, Arabian countries, female, younger age, lower education and income, having no insurance, living in rural area, self-identification as a racial/ethnic minority, belief that vaccine is not necessary, belief that natural exposure to infection gives safest protection, insufficient testing of COVID-19 vaccines, belief authorities are motivated by financial gain rather than the health of people, conspiracy beliefs, presence of chronic disease |
| 10 | Crawshaw et al. 2021 | Living behavioural science evidence synthesis (31 August 2021) | 175 | November 2020–31 August 2021 | Global | General population | Advice from medical professionals encouraging vaccination, concerns about becoming infected with COVID-19, positive attitude/high perceived benefit of COVID-19 vaccines, belief COVID-19 vaccines will help protect family, certain political preferences/identities, viewing COVID-19 vaccination as a social/collective responsibility, historical seasonal influenza vaccination, members of families/close social network having COVID-19, fear of being infected with COVID-19 and its impact, psychological distress symptoms (stress, depression, anxiety), having access to and trust in reputable information sources | Gaps in knowledge about COVID-19 vaccines, access issues in terms of time, convenience, and cost, mistrust in government/public health response to COVID-19, negative influence of close contacts and high-profile persons, direct advice from medical professionals about vaccination, concerns about COVID-19 vaccine safety, concerns about COVID-19 vaccine development, concerns about COVID-19 vaccine efficacy, concerns about COVID-19 vaccine necessity, concern about adverse reactions (specifically contraindications among patients) |
| 11 | Galanis, Vraka, Fragkou, et al. 2021 | Systematic review | 24 | Until 14 July 2021 | Global | HCWs | Being a physician, male HCW, White HCW, older age, higher educational level and outcome, work in healthcare facilities in urban areas, being HCWs with presence of chronic disease, flu vaccination during previous season, stronger vaccine confidence and positive attitude towards a COVID-19 vaccine, fear of COVID-19, individual perceived risk from COVID-19, less complacent attitude to COVID-19 | - |
| 12 | Galanis, Vraka, Siskou, et al. 2021 | Systematic review | 12 | December 2020–May 2021 | Global | General population | Native-born, White individuals, being male, older age, higher education level, higher income, higher rank occupation, working in a health-related occupation, participants without a history of COVID-19 infection, higher self-perceived COVID-19 vulnerability, better information about COVID-19 vaccines, living with others, presence of chronic disease | Concerns about safety and effectiveness of vaccines, illness, medication, pregnancy, fertility, breastfeeding, religious reasons, ethical reasons, previous COVID-19 diagnosis, self-estimation that COVID-19 is not a severe disease, negative experiences of vaccines among family members or friends |
| 13 | Garg et al. 2021 | Systematic review | 9 | July 2021 | Global | LGBTQ+ population | Altruism,  Being HIV+ LGBTQ+ Pennsylvanians | Medical mistrust and social concern regarding COVID-19 vaccine stigma, concerns to vaccine side effects, long-term safety, previous negative experiences with healthcare providers, concerns about vaccine safety and effectiveness, Black LGBTQ+ Pennsylvanians have more concern about vaccine than all other LGBTQ+ Pennsylvanians |
| 14 | Hajure et al. 2021 | Systematic review | 24 | May­­–20 July 2021 | Africa | HCWs | Older age, being male, higher education level, fear of COVID-19, trust in government, being vaccinated against seasonal flu during previous season, presence of chronic disease, history of recommendation, living with nuclear family | Concerns about safety of vaccines, presence of depression symptoms in past week, economic problems, absence of enough clinical trials, inadequate time for making decision |
| 15 | Januszek et al. 2021 | Systematic review and meta-analysis | 9 | Until 10 July 2021 | Global | Pregnant women having at least primary school education | Trust in importance and effectiveness of vaccine, explicit communication about safety of COVID-19 vaccines for pregnant women, acceptance of other vaccines such as influenza, belief in importance of mass vaccination for one's country, anxiety about COVID-19, trust in public health agencies/health science, compliance with mask guidelines | Fear of harm to foetus, and belief in side effects that could negatively affect foetus, lower socioeconomic status, younger age, lower education, suspicion that introduction of vaccines and advertising campaigns are politically motivated |
| 16 | Joshi et al. 2021 | Scoping review | 22 | 15 December 2020 | Global | General population | Male sex, high income, older age, married, high education, health insurance coverage | Parenthood, homemaker, retired, unemployed, child presence of chronic disease, younger age (<60), Black race, low educational attainment, rural settings, low income, no health insurance |
| 17 | Kamal et al. 2021 | Rapid systematic review | 21 | January 2020–May 2021 | UK | Minority ethnicities | Trust in communication and vaccine outcomes, more information about vaccine, including information about effectiveness, side effects and ingredients, proactive engagement of HCWs from diverse ethnic backgrounds, increased visibility of less well-represented groups in the media, trusted sources such as family, friends, community members and religious leaders, risk perception, previous infection, knowing people who had been unwell or died from COVID-19, concerns about infection of families and loved ones, hearing positive things about vaccine | Pre-existing mistrust of formal services, lack of knowledge and information about vaccine's safety, misinformation, complex and changing guidance, inaccessible communications, conflicting information from different sources, practical barriers such as location of vaccine centres |
| 18 | Khubchandani and Macias 2021 | Systematic review and meta-analysis | 13 | February 2020–February 2021 | US | Hispanics and African-Americans | Older age, being male, higher income and education level | Larger household size, medical mistrust, history of racial discrimination, greater exposure to myths and misinformation, perceived risk of getting infected with COVID-19, past vaccine compliance and beliefs about vaccines, concerns about safety, efficacy, and side effects of the COVID-19 vaccines |
| 19 | Li et al. 2021 | Rapid systematic review | 13 | 12 February 2021 | Global | HCWs | Being male, older HCW, being physician, or research scientist, presence of chronic disease, high income and education, being democrat/liberal, previous influenza vaccination, self-perceived risk of COVID-19 and perceived impact on health, having a higher risk of COVID-19 infection such as involvement in isolation rooms, encountering suspected or confirmed COVID-19 patients, providing direct patient care, caring for COVID-19 patients and working in clinics, more confidence, less complacency and more collective responsibility, greater work stress, wanting to protect family, self, patients, and community | Being a nurse, African-American, scepticism about rapid development of vaccine, novel and unfolding science of SARS-CoV-2 and political environment, concern for vaccine safety, efficacy, effectiveness, potential side effects, especially long-term side effects, distrust in government, regulatory authorities and public health experts, lack of knowledge and information about vaccine, trust in own immune system |
| 20 | Lin et al. 2020 | Rapid systematic review | 12 | Until 20 October 2020 | Global | General population | Being over 55 or 65 years old, white Americans>Blacks, US-made vaccines more trusted than China-made or foreign-developed, national COVID-19 infection and mortality rates, perceived risk, and severity of infection, worrying about oneself or family members getting infected, recommendation from doctor, opinion of family and friends, past inoculation history, healthcare professionals' attitudes | Lower income, uninsured, living in rural area, larger household, being female, newness of COVID-19 vaccines, fear of side effects, safety, and effectiveness, belief that vaccines are unnecessary, inadequate information, unknown/short duration of immunity, general anti-vaccine stance, concern about cost |
| 21 | Luo et al. 2021 | Systematic review and meta-analysis | 9 | 19 February 2021 | Global | HCWs | Being male, aged 30 or older, history of influenza vaccination | - |
| 22 | Moola et al. 2021 | Rapid review | 15 | 28–29 April 2021 | Low- and middle-income countries | General population | Being male, higher education (graduate and postgraduate level) and income, elevated socioeconomic status, unmarried, being HCW, safe, effective, and free vaccine, stronger recommendation from healthcare providers or an influential community member, higher trust in governments and pharmaceutical companies | Exposure to misinformation about COVID-19 vaccines and public concerns over safety of vaccines, religious beliefs, residents of slum, semi-urban, and rural areas, having occupations such as agriculture, day-labour, and homemaker, being divorced, separated, or widowed >single or unmarried, received COVID-19 information from relatives, friends or social network contact who choose not to get vaccinated, political beliefs, effectiveness and risk of severe side effects of COVID-19 vaccine, unreliability of clinical trials, high cost, importance of affordability, concern about rapid development, concerns about risk of COVID-19 exposure when seeking vaccination, perceived secrecy and inadequate communication addressing fears and concerns about COVID response, suboptimal technical/scientific communication, lack of public engagement |
| 23 | Nehal et al. 2021 | Systematic review and meta-analysis | 63 | 20 October 2020–1 March 2021 | Global | General population | Being male, older age, perceptions of benefits, access to healthcare | Perceived harms and barriers of vaccines, rumours about infertility, healthcare system distrust |
| 24 | Ochieng et al. 2021 | Scoping review-modified version of Arksey and O'Malley strategy | 71 | 2020-2021 | Global | Minority groups | Perceived benefit, number of people vaccinated, collective and personal benefit themes, perceived risk of COVID-19 | Concern about safety and efficacy of COVID-19 vaccine, pharmaceutical/ government medical mistrust, fear of short and long-term side effects, fear of needles, number of injections, for Muslims religion side effects impacting on Ramadan, fertility issues specifically in Arab female population, Black persons living with HIV concern for safety of vaccine, racism, underrepresentation in medical clinical research, biased non-diverse healthcare providers, lower educational attainment, general distrust in vaccines, robustness of conspiracy beliefs, ethnic minorities with lower level of income (Chinese, Black, Latin and others), families with children, country of manufacturing for the vaccine, cost of vaccine, the vaccine's novelty, short duration of development, duration of immunity, vaccine development outside US, particularly China, COVID-19's novel vaccination technology, information reliability, belief that the COVID-19 vaccine contains non-Halal or alcohol-based components, moderate or conservative political leanings, residents of nonmetropolitan areas, lack of past vaccine compliance, religious beliefs, belief that it is better to have natural immunity instead of vaccines |
| 25 | Robinson et al. 2021 | Systematic review and meta-analysis | 28 | January–November 2020 | Global | General population | - | Being female, younger, lower income or educational level, belonging to an ethnic minority group |
| 26 | Terry et al. 2021 | Systematic review and meta-analysis | 23 | 01 January 2020–12 February 2021 | Global | Adults from general population | Personal fear of COVID-19, positive attitudes towards past influenza vaccines, perception that vaccine reducing risk of COVID-19 infection, being male, being White, trust in scientists and government | Lower perceived individual risk and perceived severity of COVID-19, lower level of worry regarding the pandemic and lower perceived likelihood of becoming infected with COVID-19, higher levels of perceived vaccine harm, concerns about vaccine efficacy, fear of potential adverse effects of vaccine, being BAME individual, lower household income, lower educational attainment, being younger (<30/35), speed of vaccine production and lack of evidence, general anti-vaccine stance |
| 27 | Veronese et al. 2021 | Systematic review and meta-analysis | 15 | Until 18 June 2021 | Global | Older people | - | Being Hispanic in North America, lower level of education, lower income |
| 28 | Wake 2021 | Systematic review | 45 | Until 8 May 2021 | Global | General population | Male sex, White ethnicity, high education level, prior COVID-19 infection, perceived risk of COVID-19, health insurance, perceived benefit of vaccines, better information about COVID-19 vaccines, perceived efficacy and effectiveness of the COVID-19 vaccination, recommended for vaccination, political leaning (liberals), perceived severity of COVID-19, belief that vaccination relieves worry about COVID-19, believing in mandatory COVID-19 vaccination, presence of chronic disease, previously received an influenza vaccine, believing vaccines can stop pandemic, fear of COVID-19, receiving any vaccine in past five years, perception of COVID-19 can be prevented by vaccine, willingness to protect others by getting oneself vaccinated, taking direct care of a COVID-19 patient, member of household belonging to vulnerable group, believing COVID-19 virus was not developed in laboratories, believing COVID-19 is far more contagious and lethal than H1N1 virus, compliance with community mitigation strategies, being in a private sector, encounters with suspected or confirmed COVID-19 patients, believing new waves of COVID-19 are coming, knowledge about COVID-19, perception that COVID-19 will persist | Perceived vaccine barriers, perceived potential vaccine harms, having COVID-19 vaccine safety concern, belief that only people who are at risk of serious illness should be vaccinated, complacency, paying for and travelling for vaccine, mistrust in government, healthcare system, public health authorities and vaccine developers |
| 29 | Wang et al. 2021 | Systematic review and meta-analysis | 38 | Until November 2020 | Global | General population | Perceived COVID-19 infection risk, protecting oneself or others, 60 or more years old, having a college degree or higher education, White ethnicity>Black ethnicity, presence of chronic disease amongst nurses, influenza vaccination in past season, trust in government, people with greater exposure to media reports about COVID-19, liberal political leanings | Female sex, lower household income, concerns about side effects and safety, having child or children, being unemployed |
| 30 | Yasmin et al. 2021 | Systematic review | 65 | Until 17 July 2021 | US | US general population | Being male, with a college degree or higher education, being >45 years old | Being African/American or non-Hispanic Blacks, being pregnant or breastfeeding women, lower income, uncertainty about vaccine safety and side effects, religious reasons, lack of trust in the healthcare system |
| 31 | Zintel et al. 2021 | Systematic review and meta-analysis | 60 | 19 November 2020–7 January 2021 | Global | General population | Being male | - |

BAME=Black, Asian and minority ethnic, CDC=Centers for Disease Control and Prevention, FDA=Food and Drug Administration, HCWs=Healthcare workers, HIV=Human immunodeficiency virus, H1N1=Swine flu, UK=United Kingdom, US=United States, WHO=World Health Organization
