# Supplementary material for: Determinants for COVID-19 vaccine hesitancy in the general population: a systematic review of reviews
Source: Z Gesundh Wiss. 2022 Sep 19:1–17. Online ahead of print. doi: 10.1007/s10389-022-01753-9 (PMC9483252; doi:10.1007/s10389-022-01753-9)
Supplement: Supplementary file 2 — (DOCX 65 kb) [file 10389_2022_1753_MOESM2_ESM.docx]

**Title: Determinants for COVID-19 vaccine hesitancy in the general population: A systematic review of reviews**

***Journal of Public Health ISSN 1613-2238***

**Supplementary Table 2** **JBI Checklist for Systematic Review** **and Research Syntheses**

| Studies/ Questions | Q1. | Q2. | Q3. | Q4. | Q5. | Q6. | Q7. | Q8. | Q9. | Q10. | Q11. | Quality |
| --- | --- | --- | --- | --- | --- | --- | --- | --- | --- | --- | --- | --- |
| Aboelsaad et al. 2021 | Yes | Yes | Yes | Yes | Yes | Yes | Yes | Yes | Yes | Yes | No | High |
| Ackah et al. 2021 | Yes | Yes | Yes | Yes | No | No | Yes | Yes | NA | Yes | Yes | Moderate |
| Al-Amer et al. 2021 | Yes | Yes | Yes | Yes | Yes | Yes | Yes | Yes | NA | Yes | Yes | High |
| Al-Jayyousi et al. 2021 | Yes | Yes | Yes | Yes | No | No | Yes | Yes | Yes | Yes | Yes | High |
| AlShurman et al. 2021 | Yes | Yes | Yes | Yes | No | No | Yes | Yes | No | Yes | No | Moderate |
| Aw et al. 2021 | Yes | Yes | Yes | Yes | No | No | Yes | Yes | NA | Yes | Yes | Moderate |
| N. Biswas et al. 2021 | Yes | Yes | Yes | Yes | No | No | Yes | Yes | No | Yes | No | Moderate |
| R. Biswas et al. 2021 | Yes | Yes | Yes | Yes | No | No | Yes | Yes | No | Yes | Yes | Moderate |
| Cascini et al. 2021 | Yes | Yes | Yes | Yes | Yes | Yes | Yes | Yes | NA | Yes | Yes | High |
| Crawshaw et al. 2021 | Yes | Yes | Yes | Yes | No | No | Yes | Yes | NA | Yes | Yes | Moderate |
| Galanis, Vraka, Fragkou, et al. 2021 | Yes | Yes | Yes | Yes | Yes | Yes | Yes | Yes | Yes | Yes | Yes | High |
| Galanis, Vraka, Siskou, et al. 2021 | Yes | Yes | Yes | Yes | Yes | Yes | Yes | Yes | NA | Yes | Yes | High |
| Garg et al. 2021 | Yes | Yes | Yes | Yes | No | No | Yes | Yes | NA | Yes | Yes | Moderate |
| Hajure et al. 2021 | Yes | Yes | Yes | Yes | Yes | Yes | Yes | Yes | NA | Yes | Unclear | High |
| Januszek et al. 2021 | Yes | Yes | Yes | No | No | No | Yes | Yes | NA | Yes | Yes | Moderate |
| Joshi et al. 2021 | Yes | Yes | Yes | No | No | No | Yes | Yes | No | Yes | Yes | Moderate |
| Kamal et al. 2021 | Yes | Yes | Yes | Yes | Yes | Yes | Yes | Yes | NA | Yes | Yes | High |
| Khubchandani and Macias 2021 | Yes | Yes | Yes | Yes | No | No | Yes | Yes | No | Yes | No | Moderate |
| Li et al. 2021 | Yes | Yes | Yes | Yes | Yes | Yes | Yes | Yes | NA | Unclear | Yes | High |
| Lin et al. 2020 | Yes | Yes | Yes | Yes | Unclear | Unclear | Yes | Yes | No | Yes | Yes | Moderate |
| Luo et al. 2021 | Yes | Yes | Yes | Yes | Yes | Yes | Yes | Yes | Yes | Yes | Yes | High |
| Moola et al. 2021 | Yes | Yes | Yes | Yes | No | No | Yes | Yes | No | Yes | Yes | Moderate |
| Nehal et al. 2021 | Yes | Yes | Yes | No | Yes | Unclear | Yes | Yes | Yes | No | Yes | Moderate |
| Ochieng et al. 2021 | Yes | Yes | Yes | Yes | No | No | Yes | Yes | NA | No | No | Moderate |
| Robinson et al. 2021 | Yes | Yes | Yes | Yes | No | No | Yes | Yes | Yes | Yes | No | Moderate |
| Terry et al. 2021 | Yes | Yes | Yes | Yes | Yes | Yes | Yes | Yes | No | Yes | Yes | High |
| Veronese et al. 2021 | Yes | Yes | Yes | No | No | No | Yes | Yes | Yes | Yes | Yes | Moderate |
| Wake 2021 | Yes | Yes | Yes | Yes | Yes | No | No | Yes | NA | Yes | Yes | Moderate |
| Wang et al. 2021 | Yes | Yes | Yes | Yes | Yes | Yes | Yes | Yes | No | Yes | Yes | High |
| Yasmin et al. 2021 | Yes | Yes | Yes | Yes | No | No | Yes | Yes | No | Yes | Yes | Moderate |
| Zintel et al. 2021 | Yes | Yes | Yes | Yes | Yes | Yes | Yes | Yes | No | Yes | Yes | High |

NA=Not Applicable
